# Response Surface Methodology for Optimization of Genistein Content in Soy Flour and its Effect on the Antioxidant Activity

**Published:** 2018

**Authors:** Asmaa Abdella, Ashraf F. El baz, Emad Eldin Mahrous, Alaa A. Abd El Maksoud, Ibrahim A. Ibrahim, Azza R. Abdel-Monem, Shang-Tian Yang

**Affiliations:** a *Department of Industrial Biotechnology, Genetic Engineering and Biotechnology Research Institute, University of Sadat City, Sadat City, Egypt. *; b *Food Technology Research Institute, Agricultural Research Center, Giza, Egypt. *; c *Department of Pharmacognosy, Faculty of Pharmacy, Cairo University, 11562 Cairo, Egypt. *; d *William G. Lowrie Department of Chemical and Biomolecular Engineering, Ohio State University, Columbus, Ohio, USA.*

**Keywords:** *β*-Glucosidase, Biotransformation, Genistein, Response surface methodology, Antioxidant

## Abstract

Biotransformation of isoflavones glycosides into the aglycone form is essential to attain the maximum bioavailability. The factors affecting deglycosylation of genistin in soy flour using commercial *β*-glucosidase enzyme were evaluated. The presence of genistin in soy flour was confirmed by isolation through chromatographic fractionation and identification by spectral method. Two-levels Plackett-Burman design was applied and effective variables for genistein production were determined. Agitation rate, enzyme concentration, and reaction time, owing to their significant positive effect, and pH, owing to its significant negative effect, were further evaluated using Box-Behnken model. Accordingly the optimal combination of the major reaction affecting factors was “enzyme concentration, 1 IU; agitation speed, 250 rpm; reaction time, 5 h and pH 4. The concentration of genistein can be increased by 9.91 folds (from 0.8 mg/g in the non biotransformed soy flour to 7.93 mg/g in the biotransformed one) using the determined optimal combination of major reaction affecting factors. The antioxidant activity of the non biotransformed and biotransformed soy flour extracts was determined by DPPH method. It was found that biotransformation increase the antioxidant activity by two folds. The concentration causing a 50% reduction of DPPH absorbance (EC_50_) were 10 and 5 mg/mL for the non biotransformed and biotransformed soy flour extracts, respectively.

## Introduction

Among the foods eaten by humans, soybeans contain the highest level of isoflavones, which are phytoestrogen (plant-derived phenolic compounds with structural homology to human estrogen), common in leguminous plants (1). These compounds are currently heralded to offer potential natural alternative therapies for a range of hormone-dependent conditions including menopausal symptoms (2), cardiovascular disease (3), osteoporosis (4) as well as prostate, breast, and colon cancer (5). 

Soy isoflavones exist in the form of aglycones (daidzein, genistein and glycitein) and *β*-glycosides conjugates, which include the glycosides (daidzin, genistin and glycitin), malonylglycosides and acetylglycosides. The content of daidzin and genistin are high in soybeans while the aglycones (daidzein, genistein) are found in trace quantities (6).

 Numerous studies have shown that the biological effects of isoflavones are not due to the glycoside forms but mainly to their aglycones. For example, aglycone is able to bind to estrogen receptor and hence mimic estradiol functions in the human body and thus prevent certain cancers (7). The anti-cancer function of soybean isoflavones was shown to be associated with genistein, which inhibits protein tyrosine kinases and DNA topoisomerase, and binds weakly to estrogen receptors (8). It was found that early exposure to genistein enhances cell differentiation of the mammary gland, and may confer a protective effect against carcinogenesis via this process (9). Furthermore, in vitro studies using cultured human breast cancer cells indicate that genistein inhibited the growth of both estrogen receptor-negative and estrogen receptor-positive cell lines (10). Also, genistein exhibited a much greater antioxidant activity than that of genistin (11). 

 Enzymatic transformation has been known to have several advantages over chemical methods in the transformation process. These advantages include the mild reaction conditions, the high yield of hydrolysis and the reduction of the inhibitory compounds formed (12). For this reasons, we turn our think to the use of *β*-glucosidase (EC 3.2.1.21, *β*-glucoside glucohydrolase), which mainly catalyzes hydrolysis of the *β*-1,4-glycosidic linkage in various disaccharides, oligosaccharides, as well as alkyl- and aryl-*β*-D-glucosides (13). The current work is the first time to use response surface methodology in the optimization of the biotransformation condition of the major soy isoflavone (genestin) into its aglycone (genestein) in the soy flour extract by commercial *β*-gluosidase containing enzyme (celluclast BG) and to evaluate the effect of this biotransfomation on the antioxidant activity.

## Experimental

*Plant material*

 Defatted soy flour was obtained from the Agricultural Research Center (ARC), Giza, Egypt. 

*Authentic*

 Genistein standard and DPPH were purchased from sigma. Authentic sugars; arabinose, galactose, glucose, mannose, rhamnose, and xylose were obtained from E. Merck, Darmstadt, Germany. Cellobiose was obtained from Cambrian chemical. *β*-Glucosidase containing enzyme (celluclast BG) was obtained from Novozymes. 

*Isolation of soy isoflavone glycosides*

*Materials for chromatographic study*

 Pre-coated TLC plates silica gel 60 F254 (20 × 20 cm, 0.25 mm thickness) and silica gel H type 60 were obtained from E. Merck, Darmstadt, Germany. Sephadex LH-20, for column chromatography (CC) was obtained from Pharmacia Fine Chemicals AB.

*Extraction, fractionation and isolation of soy isoflavones*

 Defatted soy flour (2.5 kg) was extracted with 70% methanol (3 L × 3) on cold till exhaustion. The extract was evaporated under vacuum at 60 °C to remove the methanol. The remaining aqueous extract was lyophilized to give 37 g. Fifteen g of the lyophilized residue was subjected to vacuum liquid chromatography (15 × 5 cm) packed with silica gel-H (55 g). Gradient elution was carried out starting with hexane: chloroform (1:1), the polarity was increased by 10% stepwise addition of chloroform till 100% chloroform followed by 10% stepwise addition of ethyl acetate till 100% ethyl acetate then 5% stepwise addition of methanol till 100% methanol, to obtain 38 fractions. The obtained fractions were monitored by TLC, similar fractions were collected together, and the solvent was evaporated under reduced pressure. Fractions containing major spots were subjected to further fractionation.

 Compound 1 (20 mg, yellowish white needle crystals) and compound 2 (10 mg, yellowish white crystals) were obtained upon re-chromatography of fractions (22-27) and (28-38), respectively, on sephadex (1 × 20 cm) using methanol for elution.

*Spectral analysis of the isolated compounds*

^1^NMR analysis was measured on ^1^H-NMR (300 MHz): Varian Mercury-VX-300 spectrophotometer.

*Acid hydrolysis of the isolated glycosides*

The two isolated glycosides (2 mg) were hydrolyzed following the method of Harborne *et al*. 1975 (14). The obtained aglycones and sugars were identified by comparison with authentic using TLC. 

*Assay of the β-glucosidase enzyme*

Cellobiose substrate (0.5 mL of 0.4%) in 0.05 M citrate phosphate buffer (pH 4.8) was added to 0.5 mL of celluclast BG enzyme dissolved in 0.5 mL of 0.05 M citrate phosphate buffer (pH 4.8). Then they were incubated for 30 min at 50 ^°^C. The reaction mixture was placed in a boiling water for 5 min to stop the reaction and then immediately cooled in an ice bath. This mixture assayed by a glucose oxidase kit to determine glucose concentration. One unit of *β*-glucosidase activity was defined as the amount of enzyme that produced 1 µmol of glucose per min from cellobiose (15).

**Table 1:** Factors and testing levels for Plackett-Burman experiment for deglycosylation of genistin by the commercial *β*-glucosidase enzyme.

**Factor**	**Symbol**	**Low level (-1)**	**High level (+1)**
Ph	X1	4	7
Temp. (°C)	X2	40	60
Time (h)	X3	0.5	3
Substrate conc. (w/v)	X4	0.05	0.1
Enzyme conc. (IU/mL)	X5	0.2	0.5
Agitation (rpm)	X6	100	200
Buffer strength (M)	X7	0.05	0.1
CaCl_2_ (mM)	X8	0.5	1
MnCl_2_ (mM)	X9	0.5	1

**Table 2:** Randomized Plakett-Burman experimental design for evaluating factors influencing deglycosylation of genistin by the commercial *β*-glucosidase containing enzyme.

	**pH**	**Temp.** **(°C)**	**Time (h)**	**Substrate conc. (mg/mL)**	**Enzyme conc. (IU)**	**Agitation (rpm)**	**Buffer strength (M)**	**CaCl**_2_ ** mM**	**MnCl**_2_ ** mM**	**Genistein conc. ** **mg/g**
1	7	40	0.5	1	0.5	100	0.05	1	0.5	1.245
2	4	60	0.5	1	0.5	200	0.05	0.5	1	2.574
3	7	60	3	1	0.2	100	0.05	0.5	1	1.734
4	4	40	0.5	0.5	0.2	100	0.1	0.5	1	1.1
5	4	60	3	0.5	0.2	100	0.05	1	0.5	2.68
6	7	40	3	1	0.2	200	0.1	1	1	2.142
7	7	40	0.5	0.5	0.2	200	0.05	0.5	0.5	0.633
8	4	60	0.5	1	0.2	200	0.1	1	0.5	2.028
9	4	40	3	1	0.5	100	0.1	0.5	0.5	4.05
10	7	60	0.5	0.5	0.5	100	0.1	1	1	1.044
11	4	40	3	0.5	0.5	200	0.05	1	1	4.167
12	7	60	3	0.5	0.5	200	0.1	0.5	0.5	3.378

N.B: Starting genistein concentration is 0.8 mg/g soy flour.

**Table 3:** Sorted parameter estimates the effect of each variable on genistin deglycosylation by commercial *β*-glucosidase enzyme using Placket-Burman design

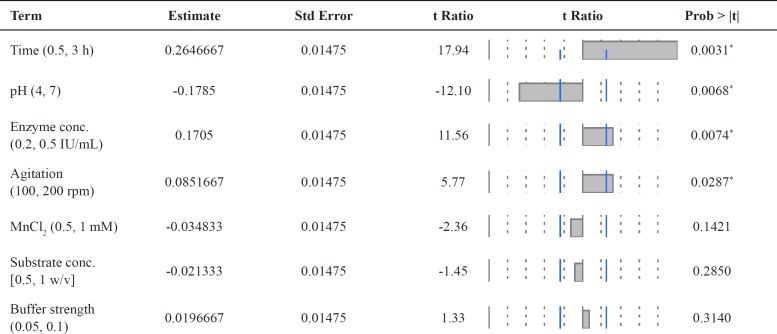

* Variables with significant effects on enzyme production.

**Table 4:** Box-Behnken factorial experimental design, representing response of genistin deglycosylation by commercial *β*-glucosidase enzyme as influenced by enzyme conc., reaction time, agitation rate and pH.

	**Pattern**	**Enzyme conc.** **(IU)**	**Agitation rate** **(rpm)**	**Time (h)**	**pH**	**Genistein** **conc.** **(mg/g)**
1	0+0+	1	250	5	4	7.93
2	+0+0	0.75	300	4	5	
3	-0+0	0.75	300	4	3	
4	0000	0.75	250	4	4	4.73
5	--00	0.5	250	4	3	4.299
6	-00+	0.75	250	5	3	7.016
7	00+-	0.75	300	3	4	2.383
8	-+00	1	250	4	3	6.64
9	+0-0	0.75	200	4	5	5.66
10	-0-0	0.75	200	4	3	4.483
11	0—0	0.5	200	4	4	4.5
12	++00	1	250	4	5	5.45
13	00++	0.75	300	5	4	
14	+00+	0.75	250	5	5	
15	0+0-	1	250	3	4	3.58
16	+00-	0.75	250	3	5	2.92
17	-00-	0.75	250	3	3	3.528
18	0-0+	0.5	250	5	4	
19	0++0	1	300	4	4	5.908
20	0-+0	0.5	300	4	4	3.946
21	0+-0	1	200	4	4	
22	+-00	0.5	250	4	5	3.348
23	00--	0.75	200	5	4	6
24	0-0-	0.5	250	3	4	2.182
25	00--	0.75	200	3	4	3.73

N.B: Starting genistein conc. is 0.8 mg/g soy flour.

**Table 5:** Sorted parameter estimates the effect of each variable on genistin deglycosylation by commercial *β*-glucosidase enzyme using Box-Behnken design.

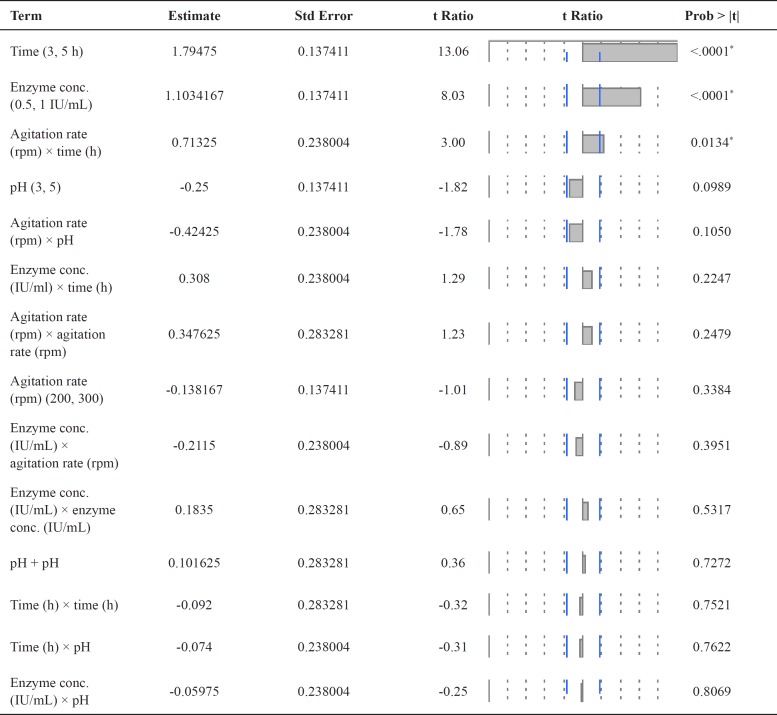

* Variables with significant effects on enzyme production.

**Figure 1:**
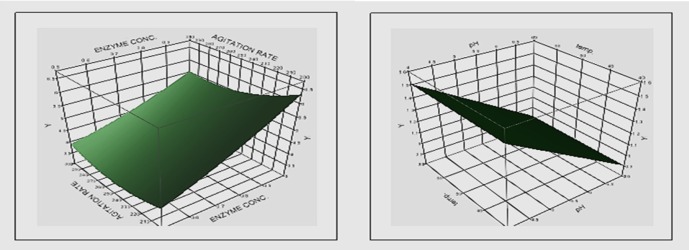
Three-dimensional surface plots showing the relationships between significant tested components and soy isoflavone aglycone yield

**Figure 2:**
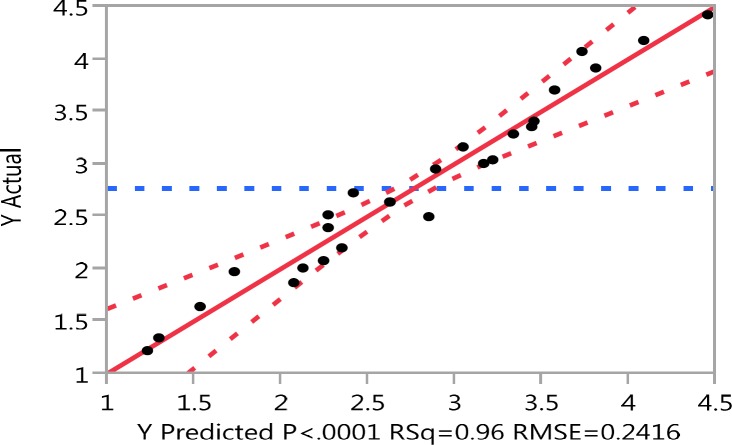
Experimental values *vs.* predicted values for genistein aglycone produced using commercial *β*-glucosidase enzyme.

**Figure 3:**
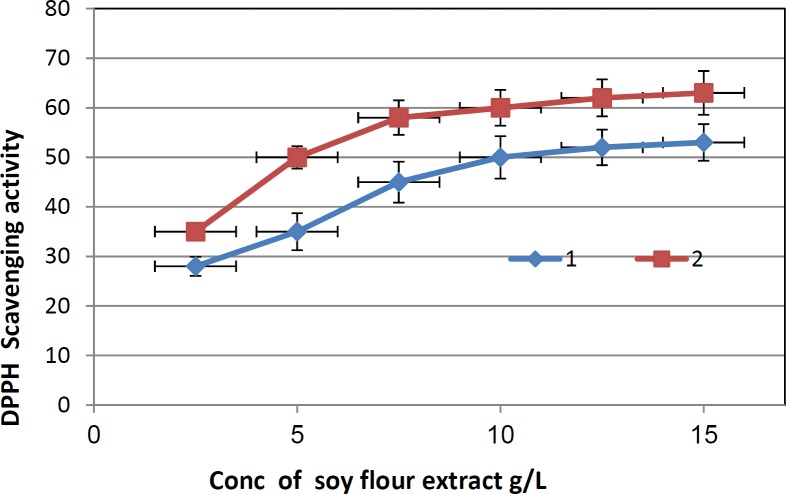
DPPH scavenging activity of the non biotransformed soy flour extract and biotransformed soy flour extract. 1: Non biotransformed soy flour extract; 2: Biotransformed soy flour extract using the commercial *β*-glucosidase containing enzyme.

*Enzymatic hydrolysis of soy flour*

Celluclast BG enzyme in citrate phosphate buffer (0.5 mL) was incubated with 0.5 mL of CaCl_2_ and 0.5 mL of MnCl_2_ at room temperature for 60 min. Then, 0.5 mL of this solution was incubated with 0.5 mL of defatted soy flour (substrate) suspended in citrate phosphate buffer under the conditions indicated in the experimental design (Tables 2 and 4). The reactions were terminated by boiling the suspension for 5 min, and then the hydrolyzed solutions were centrifuged at 11,000 rpm for 15 min to collect supernatants containing isoflavones.

*HPLC determination of genistein*

The genistein content was determined by Shimadzu HPLC system (Kyoto, Japan). Chromatographic separation was carried out on Dikma Diamonsil C18 column (4.6-250 mm Dima Co., Ltd., Orlando, FL). The mobile phase had the following composition: 0.1% (v/v) acetic acid in filtered MilliQ water (solvent A), and 0.1% (v/v) acetic acid in acetonitrile (solvent B). The following gradients for solvent B was applied: 15–25% over 35 min, 25–26.5% over 12 min, and 26.5–50% over 30 sec, followed by isocratic elution for 14.5 min. with a flow rate of 1.0 mL/min and column temperature setting at 40 °C and absorbance was measured at 254 nm (16). 

*Optimization of genistein production based on multifactorial experiments*

*Evaluation of the factors affecting genistein production*

Nine assigned variables (pH, reaction time, temperature, substrate conc., enzyme conc., agitation rate, CaCl_2_, MnCl_2_ and buffer strength) were screened in twelve experimental trials using Plackett-Burman experimental design (17). Table 1 illustrates the examined factors, as well as the levels of each factor used in the experimental design, whereas Table 2 represents the design matrix. The magnitude and the ranking of each variable in Plackett-Burman design were estimated by statistical analyses of the data. The main effect of each variable was calculated as the difference between the average of measurements made at the high value (+) and at the low value (-) (Table 3). All experiments were carried out in triplicate and the concentration of genistein aglycone (mg/g) was taken as the response (dependent variable).

*Optimization of the factors affecting enzymatic deglycosylation of genistin*

The variables with significant effects on enzyme production, as identified by the Plackett–Burman design were further optimized using a response surface Box–Behnken design (18). The design comprised 25 experiments where pH, reaction time, enzyme conc. and agitation rate were tested at three levels and in multiple combinations with the other parameters (Table 4). The effect of each variable on genistin deglycosylation was estimated by statistical method and the results were given in Table 5 and illustrated in Figure 1. The whole set of experiments was performed in triplicate and the mean response was used for analyses.

*Statistical analysis*

The optimal value for production of soy isoflavone aglycone was estimated using SAS JMP 8 NULL program tools for a regression analysis of the obtained experimental data. The quality of the fit of the polynomial model was expressed by the coefficient of determination “RSq”. The experiments were performed in triplicate and the mean values were given.

*Verification of the model*

The optimal conditions realized from the optimization experiment were verified experimentally and compared with the data calculated from the model (Figure 2). The determination coefficient (RSq) was 0.96.

*Antioxidant assay*

Antioxidant activity of soy flour methanolic extract was determined by the ability to scavenge 2,2-diphenyl-1-picryl-hydrazyl (DPPH) radicals. One mL DPPH (0.1 mM) solution in methanol was mixed with 100 µL of the crude soy extract, vortexed well, and then incubated for 30 min in the dark at room temperature and the absorbance was measured at 517 nm. For the control sample, the crude soy extract was replaced with 100 µL methanol. This antioxidant activity was given as percent (%) DPPH scavenging and calculated as [(control absorbance - extract absorbance)/(control absorbance) × 100].

Percentage of the DPPH scavenging activity was plotted versus the different concentrations of soy flour extracts used (Figure 3) and the concentration causing a 50% reduction of DPPH absorbance was taken as the half maximal inhibitory concentration (EC_50_) value. EC_50_ values were obtained by extrapolation of the data and calculated on the basis of the total solid contents of each methanolic extract (19).

## Results and Discussion

*Identification of the isolated compounds*

To confirm the presence of genistin in soy flour, it was subjected to chromatographic fractionation resulted in isolation of two compounds (1 and 2). By comparing ^1^HNMR spectral data of compounds 1 and 2 with the previously reported data (20), as well as by acid hydrolysis and comparison with authentic aglycones and sugars using TLC, these compounds were identified as genistin and daidzin, respectively. 

Compound 1: *R*_f _= 0.40 in ethyl acetate: MeOH: H_2_O: Formic acid (100: 16: 13: 20 drops), showed dull fluorescence in UV, no color with *p*-anisaldehyde spray reagent.^ 1^H-NMR (CD_3_OD): *δ*_H_ 8.15 (1H, *s*, H-2), 7.36 (2H, *d*, *J*=7.6 Hz, H-2′ and H-6′), 6.86 (2H, *d*, *J*=7.6 Hz, H-3′ and H-5′). 6.71 (1H, *d*, *J*=2.1 Hz, H-8), 6.51 (1H, *d*, *J*=2.1 Hz, H-6), 5.19 (1H, *d*, *J*=7.6 Hz, H-1^′′^), 3.43-4.12 (*m*, Sugar protons). 

Compound 2: *R*_f_ = 0.45 in ethyl acetate: MeOH: H_2_O: Formic acid (100: 16: 13: 20 drops), showed dull fluorescence in UV, no color with *p*-anisaldehyde spray reagent.^ 1^H-NMR (CD_3_OD): *δ*_H_ 8.208 (1H, *s*, H-2), 8.126 (1H, *d*, *J*=9.2 Hz, H-5), 7.358 (2H, *d*, *J*=8.4 Hz, H-2′ and H-6′), 7.223 (1H, *dd*, *J*=10.7, 1.5 Hz, H-6), 7.207 (1H, *d*, *J*=2.1 Hz, H-8), 6.846 (2H, *d*, *J*=8.4 Hz, H-3′ and H-5′), 5.029 (1H, *d*, *J*=7.6 Hz, H-1^′′^), 3.38-3.94 (*m*, Sugar protons).

*Optimization of genistein production*

A sequential optimization strategy was applied in this work, where the first phase dealt with screening and identifying the reaction mixture components and conditions affecting genistein aglycone production by the *β*-glucosidase enzymes. Once the significant factors were determined, the second phase involving ascertaining the combinations leading to the maximum genistein aglycone yield were carried out.

In the first phase, a Plackett-Burman experimental design was applied to reflect the relative importance of various enzymatic biotransformation factors. The data shown in Table 2 revealed a wide variation in genistein conc. (0.633-4.167 mg/g), thereby reflecting the significant effect of the studied factors for attaining a higher productivity. Sorted parameters estimates of the nine tested variables, revealing that enzyme conc., time and agitation rate had a significant positive effect on genistein aglycone production, whereas, pH had a significant negative effect (Table 3).

To improve the pre-optimization formula for the subsequent optimization step, the variables with a negative-effect value obtained from the Plackett-Burman design were fixed at their (-1) coded values, and the variables with a positive-effect value fixed at their (+1) coded value. To identify the optimum response region for genistein aglycone the significant independent variables [reaction time, agitation rate, enzyme concentration and pH] were further explored at three levels. Table 4 presented the design matrix for the variables, given in both coded and natural units, plus the experimental genistein aglycone production results. 

Figure 1 shows the relationships between the significant tested factors (enzyme concentration, time, agitation rate and pH) and soy isoflavone aglycone (genistein) yield. 

The results obtained (Table 5) revealed that the reaction time is the most effective factor on genistein concentration followed by the enzyme concentration. Increasing the reaction time and enzyme concentration were accompanied by increase in genistein concentration. On the other hand, increasing pH and agitation rate were followed by decrease in genistein concentration. In general, increasing the reaction time to 5 h increased genistein concentration unless the enzyme concentration was decreased to its lower limit (0.5 IU). It is noticed that increasing pH to its high limit (5) was accompanied by decrease in the genistein concentration at agitation rate of 250 and 300 rpm, meanwhile decreasing the agitation rate to its lower limit (200 rpm) reversed this effect at the same enzyme concentration (0.75 IU) and reaction time (4 h). 

The optimal levels for the factors were found to be, enzyme concentration (1 IU), time (5 h), agitation rate (250 rpm) and pH (4), where genistein concentration reach up to (7.93 mg/g). On the other hand, the lower aglycone concentration (2.182 mg/g) was obtained at experimental conditions of: enzyme concentration (0.5 IU), time (3 h), agitation rate (250 rpm), and pH (4).

Also, it was found that, the concentration of genistein aglycones in the biotransformed suspension was increased by 9.91 folds compared to the non biotransformed one. Similar results were obtained where the content of daidzein had increased by 34 folds in soybean flour suspension biotransformed by *a *thermostable* β*-glucosidase enzyme produced from Paecilomyces thermophile (21). Maitan-Alfenas *et al*. 2014 (22) found that *β*-glucosidase increased genistein content from 1.28 mg/g in the non biotransformed to 6.37 mg/g in the biotransformed soy molasses. This result also supported by Pandijtan *et al*. 2000 (23) who found that *β*-glucosidase enzyme increased genistein content from 0.028 mg/g in the non biotransformed to 0.583 mg/g in the biotransformed soy protein concentrate.

On the model level, the correlation measures for estimating the regression equation were the determination coefficient (RSq). The closer the value of RSq to 1, the better the correlation between the measured and the predicted values. In this experiment, the value of the determination coefficient (RSq) was 0.96 indicating a high degree of correlation between the experimental and predicted values that confirms the high accuracy of the model and also suggestes that the models are satisfactory and accurate. 

*Antioxidant activity*

Lately it has been reported that reactive oxygen species (ROS) are implicated in a large number of human diseases. When an imbalance between antioxidants and generation of ROS occurs, oxidative damage can occur and generate a large number of health problems such as arteriosclerosis and cancer (24). Therefore, there is increased interest focused on natural antioxidants present in foods and medicinal plants. In this study, the antioxidant properties of the non biotransformed and biotransformed soy flour extracts were evaluated by DPPH method. The two extracts showed a scavenging activity toward the DPPH radical in a dose-dependent manner (2.5-15 mg/mL). EC_50_ were 10 and 5 mg/mL for non biotransformed and biotransformed soy flour extracts, respectively. The increase in the antioxidant activity is explained by that the hydrolysis of *β*-glucosidic linkage leads to liberation of hydroxyl groups in free aglycone which might be important to the antioxidant activity of the extract (25). 

## Conclusion

The estimated optimal values for production of soy isoflavone aglycone were found to be enzyme concentration, (1 IU), time (5 h), agitation rate (250 rpm), and pH 4. The Biotransformation of soy flour by *β*-glucosidase enzyme using these optimal factors increased genistein concentration by 9.91 folds.

There was 2 fold increase in the antioxidant activities of the biotransformed soy flour suspension comparing to the non biotransformed one.
